# Functional analysis of thioredoxin from the desert lichen-forming fungus, *Endocarpon pusillum* Hedwig, reveals its role in stress tolerance

**DOI:** 10.1038/srep27184

**Published:** 2016-06-02

**Authors:** Hui Li, Jiang-Chun Wei

**Affiliations:** 1 State Key Laboratory of Mycology, Institute of Microbiology, Chinese Academy of Sciences, Beijing 100101, China; 2 University of Chinese Academy of Sciences, Beijing 100049, China

## Abstract

*Endocarpon pusillum* is a lichen-forming fungus with an outstanding stress resistance property closely related to its antioxidant system. In this study, thioredoxin (Trx), one of the main components of antioxidant defense systems in *E. pusillum* (EpTrx), was characterized and analyzed both in transgenic yeasts and *in vitro*. Our analyses identified that the heterologous expression of EpTrx in the yeast *Pichia pastoris* significantly enhanced its resistance to osmotic and oxidative stresses. Assays *in vitro* showed EpTrx acted as a disulfide reductase as well as a molecular chaperone by assembling into various polymeric structures. Upon exposure to heat-shock stress, EpTrx exhibited weaker disulfide reductase activity but stronger chaperone activity, which coincided with the switching of the protein complexes from low molecular weight forms to high molecular weight complexes. Specifically, we found that Cys31 near but not at the active site was crucial in promoting the structural and functional transitions, most likely by accelerating the formation of intermolecular disulfide bond. Transgenic *Saccharomyces cerevisiae* harboring the native EpTrx exhibited stronger tolerance to oxidative, osmotic and high temperature stresses than the corresponding yeast strain containing the mutant EpTrx (C31S). Our results provide the first molecular evidence on how Trx influences stress response in lichen-forming fungi.

Lichen represents a symbiotic life form, consisting of a mycobiont (fungal partner) and a photobiont (green algae or cyanobacteria). In this type of symbiotic associations, the photobionts obtain and supply carbohydrates through photosynthesis while the mycobionts obtain water and minerals and protect their photobionts against external stresses such as drought and UV radiation. Lichens are found in almost all biomes across the planet, and dominate in some extreme habitats such as arid deserts and polar regions^1^,^2^,^3^.

As poikilohydric organisms, lichens are able to survive in desiccated state for long period of time and quickly resume normal photosynthesis and metabolic activity within several minutes upon rehydration^4^,^5^,^6^,^7^. Such abilities have contributed to making lichens more abundant and diverse than vascular plants in arid regions^8^. Understanding the mechanisms of drought resistance in lichens could help transferring the drought resistant feature of lichens to other organisms, which should significantly enhance our ability to combat desertification in many parts of the world.

In recent years, both the morphological and biochemical features of drought resistance in lichens have been studied widely^6^,^7^,^9^,^10^,^11^,^12^,^13^,^14^,^15^,^16^. In contrast, molecular studies are relatively few and the limited studies suggest that the antioxidant system plays an important role in drought resistance^17^,^18^. For example, in the study of three lichens with different degrees of drought tolerance, the most drought resistant species was able to reversibly switch the redox status of reduced glutathione (GSH) and oxidized glutathione (GSSG) at a relative high rate during the circle of desiccation and rehydration. In contrast, the least drought resistant species failed to oxidize GSH or reduce GSSG quickly^10^. In our recent comparative transcriptome analysis of the lichen-forming fungus *E. pusillum,* a number of genes encoding proteins related to the antioxidant system were up-regulated under 20% PEG-induced dehydration stress. The genes include those that encode antioxidant enzymes and low-molecular-weight antioxidants such as glutathione and thioredoxin^18^.

Thioredoxins (Trxs) are ubiquitous oxidoreductases with a wide variety of functions in all kingdoms of life^19^. All Trxs possess a similar three-dimensional structure called Trx-fold. Each Trx-fold contains five β-strands surrounded by four short α-helices, and a conserved WCXXC catalytic motif located on the surface of the protein^20^. The two conserved redox-active Cys residues are involved in regulating the redox status of target proteins through disulfide/dithiol exchange reactions. In a redox-dependent manner, Trxs contributes to maintaining the global redox environment in cells, protecting organisms against oxidative stress, as well as participating in intracellular signaling pathways^21^,^22^. For example, in mammals, Trx participates in regulating the ASK1 MAPK pathway by changing its redox status. The reduced Trx interacts with apoptosis signal regulating kinase1 (ASK1) by disulfide bonding to inhibit the activity of ASK1. However, after oxidized by ROS, Trx forms intramolecular disulfide bonds between the two active Cys residues and releases from ASK1, which brings about a free active ASK1 to induce apoptosis^21^,^23^,^24^. Apart from these functions, Trxs also play a role in assembling the T7 DNA polymerase complex and protecting proteins from denaturation under external stresses in a redox-independent way^25^,^26^.

Trx was initially considered as a disulfide reductase. However, several recent studies have also noted the chaperone activity of Trx from a variety of organisms. The molecular chaperone activity of Trx was first reported in *E. coli*, as Trx contributed to the correct refolding of citrate synthase in a concentration-dependent rather than a redox-dependent manner^27^. Since then, the two functions of Trx have been studied intensively, especially in plants. In *Arabidopsis thaliana*, a cytoplasmic Trx, AtTrx-h3, was found to act as both a disulfide reductase and a molecular chaperone. Interestingly, the two Cys residues at the active sites were found essential for the reductase function but not for the molecular chaperone activity. The disulfide reductase activity was observed mainly in its low molecular weight (LMW) form such as the monomeric and dimeric forms. In contrast, the chaperon activity predominates in the high molecular weight (HMW), homo-multimeric forms. The structural switching from LMW to HMW forms is accompanied by the functional change from a reductase to chaperone, leading to enhanced tolerance to external stresses^26^,^28^. Similarly, in *Nicotiana tabacum*, two plastid Trxs possess both functions and their relative importance were regulated by its oligomeric structures^29^. Several other Trx-fold-containing proteins such as the protein disulfide isomerase (PDI), 2-Cys peroxiredoxin (Prx) and Trx reductase (TR) all showed a molecular chaperone function^27^,^30^,^31^,^32^. Although Trx in several model organisms has been investigated, there is currently limited information on Trx from organisms living in extreme habitats. Thus the function of Trx in these stress-resistant organisms remains to be identified.

*Endocarpon pusillum* Hedwig, a dominant species in the Tengger desert of China, is one of the most drought-resistant organisms known. The pure fungal culture of *E. pusillum* without its phycobiont *Diplosphaera chodatii* Bialosuknia, can survive for up to seven months under both desiccation and starvation stresses. In contrast, its phycobiont *D. chodatii* can survive up to two months under a similar desiccation stress but not under both desiccation and starvation stresses^16^. The results of this study suggest that the mycobiont in *E. pusillum* is more drought-resistant than the phycobiont and as such, the mycobiont likely plays a greater role than the phycobiont in this lichen’s drought resistance in nature.

In the present study, we characterized the disulfide reductase and chaperone activities of the single Trx protein in the mycobiont of *E. pusillum* through heterologous expression, and analyzed its effect on stress resistance in transgenic yeasts. Using site-directed mutagenesis, we also studied the role of the additional Cys residue of EpTrx outside the active site, and demonstrated its crucial role in protein functional and structural switching.

## Results

### Transcriptional level of *Eptrx* gene is induced by PEG

**Figure 1:**
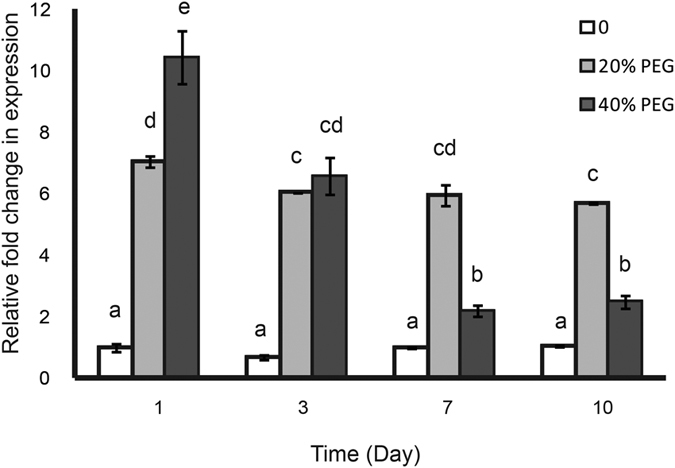
Transcriptional level of *Eptrx* gene induced by PEG. The relative expression of *Eptrx* (standardized to that of *EF-1α* gene) under 20% or 40% PEG was measured at various time points with respect to the control (without PEG treatment at day 1). The statistical analysis was performed by one-way ANOVA with the LSD *t* test to determine whether the observed differences were statistically significant (p < 0.01).

Polyethylene glycol (PEG) is a non-permeable osmolyte to create water deficit and used to stimulate drought environment of plant or fungi^18^,^33^,^34^. The comparative transcriptome analysis of our previous work indicated the expression of *Eptrx* was up-regulated with the treatment of 20% PEG for 21 days^18^. In this study, we further analyzed the transcript levels of *Eptrx* under PEG-induced drought stress condition by quantitative RT-PCR (Fig. 1). To our knowledge, 20% and 40% PEG treatment provide an adaptable and seriously stressful environment, respectively. Thus we detected the related expression levels of the *Eptrx* gene under 20% and 40% PEG-induced stress at various time points. The expression of *Eptrx* in the mycobiont cultured without treatment of PEG was used as a reference negative control. On the first day, the expression level of *Eptrx* was up-regulated to 7.1 and 10.4 folds under 20% and 40% PEG treatment, respectively. After 3 days of incubation, the transcriptional level of *Eptrx* in the 20% PEG treatment declined slightly to about 6 folds higher than the negative control and this level was maintained until at least day 10. In contrast, the transcript level in the 40% PEG treatment declined significantly at both day 3 and day 7. This difference might be caused by the cell toxicity induced by the high concentration of PEG in culture medium. Nonetheless, the transcript levels in the day 7 and day 10 treatments were still significantly higher than the negative control. This result showed that the expression of *Eptrx* gene was strongly induced under drought-stress conditions in *E. pusillum*.

### Overexpression of *Eptrx* enhances osmotic and oxidative stresses in yeasts

**Figure 2:**
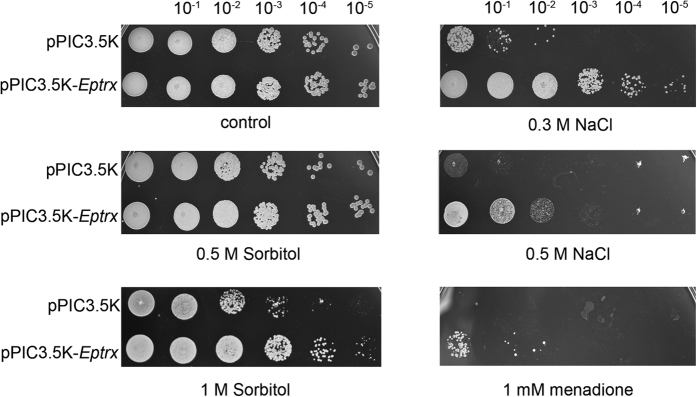
Viability of yeasts harboring empty pPIC3.5K or pPIC3.5K-*Eptrx* under osmotic or oxidative condition. Strains were adjust to OD_600_ = 0.5 and serially diluted ten-folds to 10^−1^, 10^−2^, 10^−3^, 10^−4^ and 10^−5^ in BMMY. And 4 μl of each dilutions were spotted on plates and incubated for 3 days at 30 °C. The control is on plate without any stress.

Drought environment generally creates complex stresses. EpTrx induced by PEG might also play a role in osmotic or oxidative stress tolerance. Because of lacking an efficient genetic tool in lichen-forming fungi, we studied the physiological function of EpTrx *in vivo* through the yeast expression system. EpTrx was transformed and overexpressed in *Pichia pastoris* GS115 strain driven by the AOX1 promoter, which is inducible by methyl alcohol. Ten-fold serially diluted cultures of *P. pastoris* cells transformed with the empty plasmid pPIC3.5 K or the plasmid carrying *Eptrx* were spread onto plates containing various concentrations of NaCl, sorbitol or menadione, which were respectively used to stimulate environments with osmotic, drought or oxidative stress^35^,^36^,^37^. The empty vector was used as negative control. Results showed that *Eptrx*-transformed yeasts had stronger vitality than empty-vector tansformed yeasts under 0.3 M NaCl, 0.5 M NaCl, 1 M sorbitol and 1 mM menadione (Fig. 2).

### EpTrx acts as both disulfide reductase and molecular chaperone

**Figure 3:**
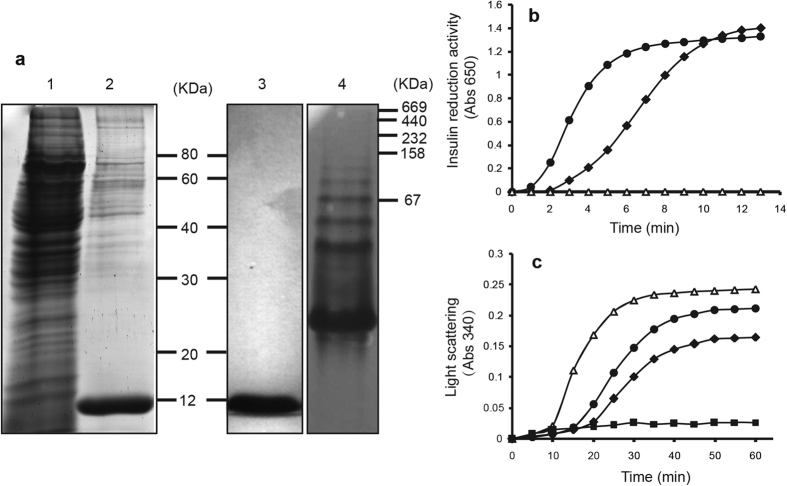
Electrophoresis analyses and dual functional identification of EpTrx. (**a**) SDS-PAGE and Native PAGE analysis of EpTrx. *Lane 1* total proteins extracted from *P. pastoris* GS115 control; *lane 2* proteins secreted to extracellular induced with 1% methanol; *lane 3* the purified EpTrx by Ni-NTA affinity chromatography on reducing SDS-PAGE gel; *lane 4* the purified EpTrx on native PAGE gel. (**b**) Disulfide reductase activity of EpTrx. Reduction of disulfide bonds using 0.75 mg insulin in the presence of 1 mM DTT. Concentrations of EpTrx used were 5 μM (♦) and 10 μM (●). 10 μM BSA (∆) instead of EpTrx was used as the negative control. (**c**) Holdase chaperone activity of EpTrx. Thermal aggregation of 2 μM MDH was examined at 43 °C, with the molar ratios of EpTrx to MDH set at 1:1 (●), 2:1 (♦) and 4:1 (■). 8 μM BSA (∆) was used as the negative control.

To confirm the function of EpTrx *in vitro*, the recombinant EpTrx protein was overexpressed in *P. pastoris* by secretory expression system. A highly purified preparation of this protein was obtained from the supernatant of liquid medium and by Ni-NTA affinity chromatography subsequently. Reducing SDS-PAGE analysis showed the expected molecular mass of the purified protein at 11.8 kDa. In addition, non-denaturing gel electrophoresis indicated that besides the monomeric form, the purified protein formed a defined homo-oligomeric complex including dimeric, trimeric and higher oligomeric forms (Fig. 3a).

The disulfide reductase activity was determined using the turbidimetric insulin precipitation assay^38^. In the presence of DTT, the insulin β-chain was reduced by Trx to show maximum absorption at 650 nm. In this assay, EpTrx accelerated the reduction of insulin in a concentration-dependent manner, for 10 μM EpTrx showed obviously higher activity than 5 μM EpTrx (Fig. 3b). Chaperone activity was measured by analyzing the ability of EpTrx to inhibit the thermal aggregation of malate dehydrogenase (MDH) which was a model substrate used to study other molecular chaperones^30^,^39^. We found EpTrx could protect the MDH from thermal aggregation effectively. In an hour, the aggregation of MDH was decreased 13% and 23% by treatment with a molar ratio of 1:1 and 2:1 (EpTrx:MDH), respectively. And aggregation was almost completely prevented at a molar ratio of 4EpTrx:1MDH (Fig. 3c). Neither disulfide reductase nor molecular chaperone activity was detected when Trx was replaced by BSA, suggesting that both activities required functional EpTrx.

### Multiple sequences analyses of Trxs indicates a specific Cys in EpTrx

**Figure 4:**
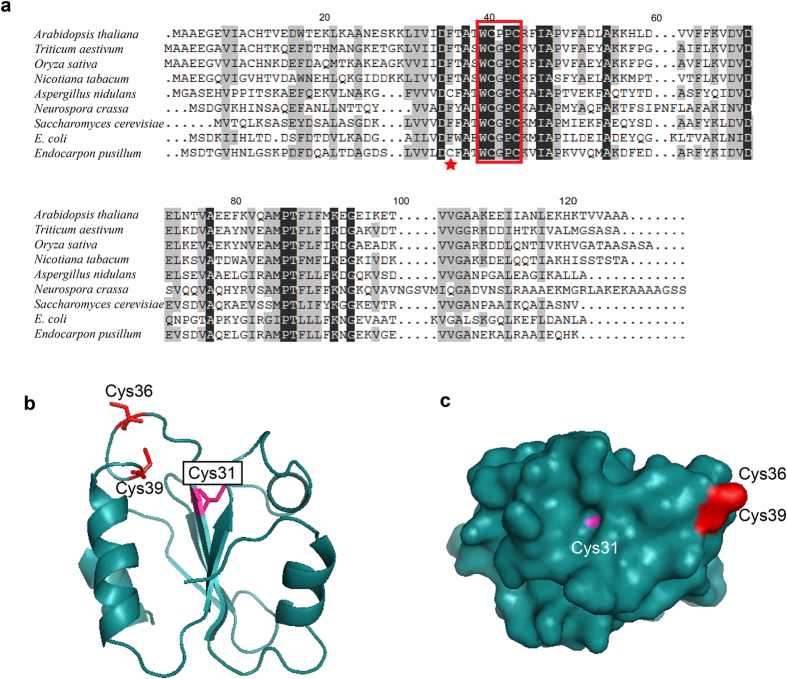
Homologous and modeling analysis of EpTrx. (**a**) Alignment of deduced amino acid sequences of the selected Trxs. Identical amino acid residues (100%) are shaded black and similar ones (>50%) are shaded gray. The conserved active site was indicated with the red box. The additional Cys was labeled by the red star. (**b**,**c**) The predicted three-dimensional structure of EpTrx constructed by the homology-based modeling. Cartoon and surface representation of EpTrx structure displaying the active-site Cys36 and Cys39 in red and the additional Cys31 in pink.

The nucleotide sequence of *Eptrx* has an open reading frame of 330 nucleotides. Alignment of the deduced EpTrx amino acid sequence with other related Trx sequences revealed that Trx is conserved through evolution. Trx from *E. pusillum* showed about 30% to 50% amino acid sequence similarity (Supplementary Table S1) to the others and the active site was highly conserved. In addition to the two conserved Cys residues at the active site, EpTrx contains an additional Cys (Cys31) closed to the active site, whereas the counterpart of Cys31 in all the other species except *Aspergillus nidulans* was Phe (Fig. 4a). Furthermore, our modeling analyses showed that Cys31 was located at the end of a β–sheet and close to the surface of protein (Fig. 4b,c). Therefore, we selected this additional Cys residue for targeted site-directed mutagenesis to investigate its potential structural and functional roles in EpTrx.

### Structural and functional switching of EpTrx dependents on Cys31

**Figure 5:**
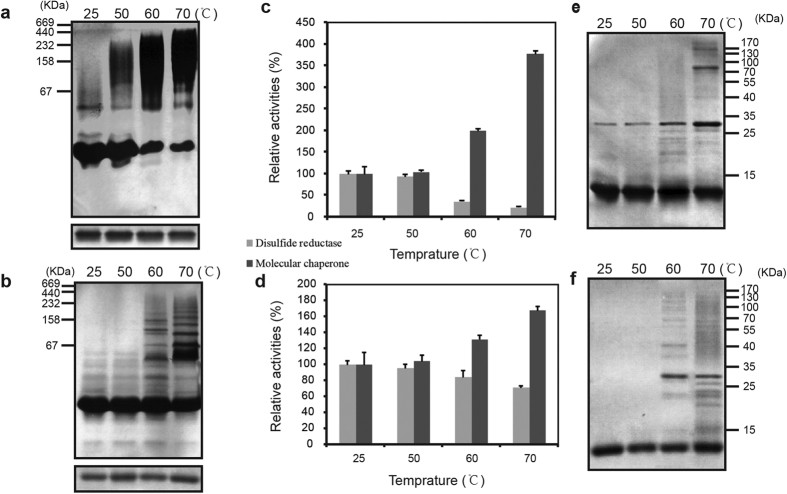
Heat shock induced structural and functional switching of EpTrx and EpTrxC31S. (**a**,**b**,**e**,**f**) Changes of oligomeric status in response to various heat shock treatments for 30 min in EpTrx (**a** and **e**) and EpTrxC31S (**b**,**f**), respectively. The oligomeric status was analyzed by 12% native PAGE gel (**a**,**b**, upper), 12% reducing SDS-PAGE gel (**a**,**b**, lower), or 12% non-reducing SDS-PAGE gel (**e**,**f**). (**c**,**d**) Relative disulfide reductase and molecular chaperone activity of heat-treated EpTrx and EpTrxC31S, respectively. Activities of the proteins were compared with those of native EpTrx or the EpTrxC31S incubated at 25 °C. Their activities at 25 °C were set to 100%.

Given that most chaperones, like small heat shock proteins, could switch their structure under heat shock^40^. And Trx-h3 from *A. thaliana* has been reported to change its structure and function under heat shock stress *in vitro*^26^. We examined whether native EpTrx or mutant EpTrx were regulated similarly. To measure the heat shock-mediated stability, the purified wild type EpTrx and the Cys31Ser mutant (EpTrxC31S) were incubated in a variety of temperature environments (25 °C, 50 °C, 60 °C and 70 °C) for 30 min. For the native EpTrx, the concentration of HMW complexes increased significantly as the temperatures rose, with a concomitant decrease in LMW forms (Fig. 5a). In contrast, the changes of oligomeric status of the EpTrxC31S were less distinctive under the same conditions, for there was no obvious smear strip on the region of HMW forms (Fig. 5b). All of the samples ran as a single protein band under reducing SDS-PAGE (Fig. 5a,b, lower), which supported that the polymeric complexes of EpTrx were homomultimers containing variable copies of the monomer.

While protein oligomerization happened, the predominant function *in vitro* might also change. Here, the activities of native EpTrx or EpTrxC31S incubated at 25 °C were set to 100%. The relative chaperone activity of the wild type protein rapidly increased to 2 folds and 3.7 folds after incubated at 60 °C and 70 °C, respectively, whereas the relative disulfide reductase activity simultaneously declined to 35% and 22% (Fig. 5c). However, the heat shock-induced functional variation tendency became obviously weakened in the EpTrxC31S, likely reflecting the structural changes caused by the C31S mutation. The chaperone activity only increased to 1.67 fold with temperature rising to 70 °C, while disulfide activity decreased to 70% (Fig. 5d). These observations together suggest that the two functions of EpTrx are regulated by the degree of polymerization and that Cys31 plays a key role in promoting the structural and functional switching upon heat shock stress.

To confirm the role of the Cys31 in structural changes of EpTrx during stress response, EpTrx was electrophoresed by non-reducing SDS-PAGE. The wild type EpTrx forms a dimer at room temperature or at 50 °C for 30 min, and the concentration of HMW forms increased with rising temperatures (Fig. 5e). However, the EpTrxC31S could not form dimeric or oligomeric structures until the temperature rose to 60 °C (Fig. 5f). The results are consistent with the hypothesis that Cys31 participates in promoting structural switching by accelerating the formation of inter-disulfide-bonded linkage.

### Yeasts transformed with native EpTrx are more stress-resistant

**Figure 6:**
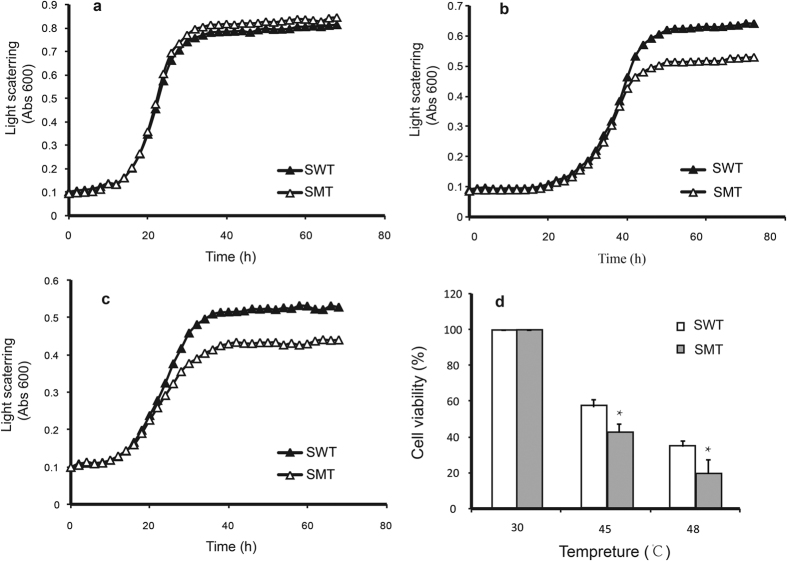
Growth curves and viability of transgenic yeast cells under various stresses. (**a–c**) The growth curves of yeasts transformed with either the wild EpTrx (SWT) or the mutant type (SMT) in normal medium containing galactose and raffinose as inducer (**a**), oxidative stress inducing medium with 2 mM H_2_O_2_ (**b**) and drought stress inducing medium with 20% PEG (**c**), respectively. The initial OD was adjusted to A_600_ = 0.1 ± 0.01, and the OD was measured every two hours. (**d**) The survival rate of transgenic yeasts (SWT and SMT) after heat shock. The cell viability was measured based on the colony-forming units after incubated at 45 °C or 48 °C for one hour. Asterisks indicate a significant difference by one-way ANOVA with the t test (P < 0.05). The colony-forming units at 30 °C was used as a reference control (100%).

To evaluate whether Cys31 affects the function of EpTrx *in vivo*, the wild type EpTrx and the mutated gene EpTrxC31S were transformed into *S. cerevisiae* (as SWT and SMT) under the control of the Gal1 promoter. The growth and viability of yeasts under various stresses were investigated. As shown in Fig. 6, in the optimal growth condition without any stress, the growth patterns of SWT and SMT are similar with the final OD_600_ values reaching 0.85 (Fig. 6a). When exposed to 2 mM H_2_O_2_, although both SWT and SMT cells increased at almost the same rate within the first 40 hours, SWT cells had a longer log phase to reach a higher OD value at stationary phase than SMT cells (Fig. 6b). And upon 20% PEG stress, during the log phase, the growth rate of SWT was higher than SMT cells (Fig. 6c). In heat-shock stress conditions, after incubating at 45 °C or 48 °C for one hour, the survival of SWT cells were 57.2% or 34.8% respectively, whereas the viability of SMT was significantly lower, decreased to 42.8% or 19.9% respectively (Fig. 6d). These data suggested that yeasts transformed with native EpTrx had stronger tolerance to oxidative, osmotic and high temperature stresses and the increased tolerance was caused by the contribution of Cys31. Considering EpTrx enhanced the stress tolerance in *P. pastoris* as well, we conclude that EpTrx is likely pivotal in protecting organisms against abiotic stress in fungi.

## Discussion

In this study, we conducted the first functional analysis of a stress related protein from lichen-forming fungi *in vivo* and *in vitro*. Even though lichens are widely regarded as among the extremely stress tolerant organisms^41^,^42^,^43^, studies on the molecular mechanism and genetic resources of lichens are very limited, partly due to their slow growth rate or our inability to cultivate most of them in pure culture^44^. Unlike most lichens, the symbiotic partners of *E. pusillum* can be cultured in the lab, making it an ideal lichen to study the two organisms separately at the molecular level. Compared with the photobiont whose main function is providing carbon resources, the mycobiont contributes to protect the photobiont against external stresses^11^,^45^. Accordingly, the adverse environmental stress on the lichen thallus can be simulated and studied by stress on the mycobiont. Indeed, the isolated mycobiont *E. pusillum* has been used in researching lichen stress resistance physiology and molecular mechanisms^16^,^18^.

In our previous work on the comparative transcriptome analysis of *E. pusillum*, the expression of *Eptrx* was found up-regulated by 20% PEG^18^. In the current study, we further explored the underlying mechanism. Under PEG-induced stress condition, the EpTrx expression showed almost 10 folds up-regulation, consistent with the earlier observation. However, sustained 40% PEG might be too overwhelming a stress for the mycobiont, leading to a slow metabolism and a decreasing level of *EpTrx* expression. Furthermore, the function of EpTrx was not limited to *E. pusillum*. When we introduced the *Eptrx* gene into *P. pastoris* and treated with NaCl, sorbitol and menadione, transformants displayed significantly enhanced resistance to osmotic and oxidative stresses. Our results suggest that the findings obtained here have significant practical implications for EpTrx.

After the function of EpTrx in stress response in both the lichen mycobiont and in *P. pastoris* was identified, we further investigated the function and activity of EpTrx *in vitro*. To our knowledge, the major role of Trx is catalyzing post-translational redox modification on target proteins by forming or disrupting disulfide bonds, which depends on two surface-exposed Cys residues^19^,^46^. Moreover, several observations have reported an additional activity of inhibiting proteins aggregation and promoting protein folding as a molecular chaperone. And the chaperone function is independent from oxidoreductase activity^27^,^47^. In this study, our results suggested that EpTrx functioned both as a disulfide reductase and a molecular chaperone, similar to that have reported in plants^26^. In addition, the dual functions of EpTrx have close association with protein oligomeric status that switch from LMW to HMW complexes induced by heat-shock stress. Indeed, this type of oligomeric structure regulated protein function have also reported in other protein. The 2-Cys Prx of *S. cerevisiae* controls its function from peroxidase to molecular chaperone according to oligomeric state in a heat-shock and redox dependent manner^30^. Similarly, a Trx-like protein (TDX) and thioredoxin reductase type C (NTRC) from *A. thaliana* are shown redox-dependent functional and structural transition^48^,^49^,^50^. By acquiring a second function and being able to reversibly switch between the two functions, such a protein could be effectively involved in responding to different stresses.

Trx is present in all organisms in at least one isotype and their amino acid sequences are evolutionally conserved from bacteria to mammalian cells. Most of the amino acids around the active sites are highly conserved and essential in disulfide reductase activity. The conserved Asp30 has been demonstrated to play a crucial role in enhancing the ionization of the thiol group at the active site^51^,^52^. Another conserved residue, Trp35, is involved in the WCXXC catalytic motif. Replacement the Trp35 by Ala or His residue leads to a strong decrease of the reductase activity^53^. The Phe31 is also highly conserved and has been reported the function of stabilizing the internal hydrophobic structure of protein^54^,^55^. However, our sequence analyses indicated a specific non-active Cys31 differed from Phe31 in the other organisms. The Cys31 located at the surface of EpTrx, and its sulfhydryl group might be involved in protein-protein interaction. To demonstrate the hypothesis, we tested the activity of a site-directed mutation at Cys31 from EpTrx. We confirmed that Cys31 stimulated the forming of oligomers with a high chaperone activity. A further non-reducing SDS-PAGE electrophoresis indicated that Cys31 influenced the formation of disulfide bonds. Therefore, the specific Cys31 of EpTrx indeed plays a pivotal role in promoting structural and functional switching by developing disulfide bonds. In tobacco, a non-active site Cys residue of the tobacco plastid Trx-f (Cys71) influenced the oligomeric status and function similarly, whereas neither the Cys19 of Trx-f nor Cys107 of Trx-m showed any effect^29^. In other organisms such as *Pisum sativum* and *Spinacia oleracea*, their Cys residues not located within the active sites (Cys12 of Trx-h1 and Cys60 of Trx-f in *P. sativum*, and Cys73 of Trx-f in *S. oleracea*) are also involved in dimerization of their respective Trx^56^,^57^.

We observed that the native EpTrx and the mutant type differed greatly under heat shock *in vitro,* a result supported the role of Cys31 in stress resistance of yeasts *in vivo*. Yeasts transformed with native EpTrx had stronger tolerance to oxidative, drought and high temperature stresses and that the enhanced abilities were likely due to the formation of HMW complexes and its greater molecular chaperone activity. These results are consistent with what has been observed with *A. thaliana* AtTrx-h3, AtTDX, and *S. cerevisiae* cPrx, as the holdase chaperone activity is responsible for the increased tolerance to heat shock or oxidative stress^26^,^30^,^48^. For example, mutant the two active Cys of NTRC from *A. thaliana* led the disulfide reductase almost completely inactive, however, the transgenic *A. thaliana* with the mutant NTRC showed only a slightly different degree of thermotolerance compared with that of wild NTRC plants, which due to the chaperone activity of mutant NTRC was nearly unchanged^39^. For desert organisms such as the lichen analyzed here, it is essential to possess a strong resistance or tolerance against dehydration and high temperature. Our results suggest that Cys31 in EpTrx from *E. pusillum* likely has been positively selected and plays an essential role for its survival in its native habitat.

In conclusion, we performed the first functional analysis of Trx from lichen-forming fungi using both *in vivo* and *in vitro* models. Our analyses identified that Trx in *E. pusillum* was involved in PEG-induced stress response significantly. The transgenic *P. pastoris* containing EpTrx showed enhanced resistance to both osmotic and oxidative stresses. *In vitro*, EpTrx possessed two functions as both a disulfide reductase and a molecular chaperone and the relative importance of these two activities was related to its oligomeric status. Under heat shock stress, Cys31 was found to be critical for accelerating the structural and functional transitions to enhance the stress tolerance of organisms. In addition, as a stress protein containing a particular functional amino acid residue and participating anti-stress in yeasts, EpTrx perhaps can also contribute to enhance the stress tolerance of plants in desert environments.

## Methods

### Fungal strain and cultivation

The lichen-forming fungus was isolated from lichen *E. pusillum* collected from Tengger Desert in China (37°32′N, 105°02′E). The pure cultured mycobiont was maintained on potato dextrose agar (PDA) slants at 4 °C. The mycelia were grown on PDA liquid media at 20 °C and 140 rpm for 21 days before they were subjected for analyses.

### Quantitative analysis of EpTrx transcription

Fresh mycelial pellet was mashed and then incubated into PDA medium containing three different concentrations of PEG (0%, 20%, and 40%) to simulate drought stress. Each treatment had four repeats. The total RNA of each culture was extracted after cultivation of 1, 3, 7 and 10 days, respectively. A pair of primers specific for the *Eptrx* gene was used to amplify a 192 bp fragment. The *EF-1α* gene was used as the internal control for quantitative RT-PCR. Primers used in quantitative analysis were listed in Table S2. The 25 μl reaction included 12.5 μl 2× Takara SYBR premix Ex Taq^TM^, 2 μl diluted cDNA, 0.5 μl of each primer (10 pmol/μl), and 9.5 μl ddH_2_O. The qRT-PCR was carried out by Bio Rad (CFX93) and performed using the following program: initial denaturation of 30 s at 95 °C, followed by 40 amplification cycles of 5 s at 95 °C, 30 s at 60 °C, 30 s at 72 °C. The comparative C_T_ method (2^−ΔΔCt^) was used for determine the expression level of *Eptrx*. The expression levels of *Eptrx* were statistically analyzed by one-way ANOVA with LSD *t* test and the result with *P* value less than 0.01 was considered statistically significant.

### Viability of yeasts under osmotic and oxidative stress

To determine the function of EpTrx *in vivo*, the complete gene encoding *Eptrx* was cloned into pPIC3.5K vector (Invitrogen) and transformed into yeast *Pichia pastoris* strain GS115 (Invitrogen). The recombinant yeasts carrying pPIC3.5K-*Eptrx* or the empty vector were cultured and induced by 1% methanol for 3 days, and adjusted OD_600_ to 0.5. Then the yeasts were diluted through ten-fold serial dilution, and 4 μl of each dilution was spotted on plates supplemented with various concentrations of NaCl, sorbitol or menadione.

### Bioinformatic analysis of EpTrx sequences

The nucleotide sequence of EpTrx was obtained from the complete genome sequence data^58^, and its amino acid sequence was analyzed using DNAMAN. The three dimensional structure of Trx was predicted using SWISS-MODEL (http://swissmodel.expasy.org/) and software PyMOL 0.99rc6 (DeLano Scientific LLC, USA). The 3D structure of Trx from *Saccharomyces cerevisiae* (PDB number 2HSY) was used as a structural template. Sequences of Trx from other organisms were downloaded from Genbank for comparison (Supplementary Table S1).

### Expression and purification of recombinant EpTrx in *P. pastoris*

The gene coding for EpTrx was cloned from cDNA of *E. pusillum* mycobiont. Sequences of primers were described in Supplementary Table S2. The amplified DNA fragment was digested with *EcoR* I and *Not* I, cloned into pPIC9K expression vector (Invitrogen) and transformed into *E. coli* DH5α for replication. After linearization with *Sac* I, the resulting extracted recombinant plasmids were introduced into *P. pastoris* strain GS115 by electro transformation. Transformants grown on Minimal Dextrose medium (1.34% YNB, 4 × 10^−5^% biotin, 2% dextrose) were collected and spread onto YPD plates containing 1, 2, 3, or 4 mg/ml G418 to select strains containing multiple copies of the plasmid. The selected transformant was cultured in BMGY (1.34% YNB, 1% Yeast Extract, 2% Peptone, 10 mM potassium phosphate buffer pH 6.0, 4 × 10^−5^% biotin and 1% glycerol) media at 30 °C until the OD_600_ reached to 1–2. Cells were harvested by centrifugation at 3000 g for 5 min, and resuspended into BMMY (0.5% methanol instead of 1% glycerol) media. Methanol was added every 24 hours to 1% (v/v) final concentration for induction of *Eptrx* expression. After 72 hours, the culture supernatant was harvested.

The target recombinant protein was purified through Ni-NTA affinity chromatography following the manufacturer’s instructions. The purified protein was detected by 12% SDS-PAGE gel and 12% native PAGE gel.

### Determination of disulfide reductase and chaperone activities

The disulfide reductase activity of EpTrx was measured by using insulin with 1 mM DTT as the reducing agent^38^. Reactions were initiated by the addition of 1 mM DTT to the reaction mixture containing 0.1 M PBS (pH 7.0), 1 mg/ml insulin (Sigma-Aldrich) and 5 or 10 μM of purified EpTrx in a final volume of 0.75 ml. The disulfide reductase activity was determined by measuring the increase in turbidity at 650 nm using 2800UV/VIS spectrophotometer (UNICO).

The chaperone activity of EpTrx was measured using the thermal aggregation of malate dehydrogenase (MDH) from porcine heart (Sigma-Aldrich) as described^30^,^48^. MDH was incubated in a 50 mM HEPES-KOH (pH 8.0) buffer at 43 °C with various concentrations of EpTrx (1:1, 2:1 and 4:1, Trx:MDH molar ratio). The change of turbidity at 340 nm was monitored with a temperature-controlled spectrophotometer (DU-800, Beckman Coulter).

### Site-directed mutagenesis of EpTrx

Site-directed mutagenesis was performed by PCR using primers shown in Table S2 in order to replace Cys31 with Ser. The PCR product was cloned into pPIC9K vector, transformed into *E. coli* and sequenced to check for the correct mutations. The resulting plasmids were introduced into *P. pastoris*. The over-expressed mutant protein was purified and its activity measured following procedures described above.

### Growth kinetics and viability of transgenic yeast cells to various stresses

The PCR products of *Eptrx* and its mutant form were cloned to vector pYES2.0 containing the URA3 marker and then transformed into *S. cerevisiea* BY4741 under the control of the Gal1 promoter. The primer sequences are shown in Table S2. To analyze the growth kinetics of the transgenic yeasts, strains were cultured in Ura Minus Media (FunGenome) plus 2% (w/v) glucose for 24 hours, and transferred to stress-inducing media with the final cell concentration adjusted to OD_600_ = 0.1 ± 0.01. The stress-inducing media contained 2% galactose and 1% raffinose instead of glucose with or without 2 mM H_2_O_2_ (for oxidative stress) or 20% PEG (for osmotic stress). The growths of the strains in each condition were measured every two hours by the Bioscreen C system. To evaluate the survival of yeasts under heat shock stress, fresh strains were transferred to the induced media to grow for 24 hours, and cultivated at 30, 45 or 48 °C for one hour. At the specific time points, 50 μl yeast cells from each treatment were spread onto YEPD agar medium. The plates were then incubated for 2 days at 30 °C, after which colonies were counted and the survival rate calculated. Amounts of cells incubated without any stress at 30 °C was set as 100%. Three repeats were performed for each treatment.

## Additional Information

**How to cite this article**: Li, H. and Wei, J.-C. Functional analysis of thioredoxin from the desert lichen-forming fungus, *Endocarpon pusillum* Hedwig, reveals its role in stress tolerance. *Sci. Rep.* **6**, 27184; doi: 10.1038/srep27184 (2016).

## Supplementary Material

Supplementary Information
