# Supplementary material for: Functional analysis of thioredoxin from the desert lichen-forming fungus, Endocarpon pusillum Hedwig, reveals its role in stress tolerance
Source: Sci Rep. 2016 Jun 2;6:27184. doi: 10.1038/srep27184 (PMC4890037; doi:10.1038/srep27184)
Supplement: Supplementary Information [file srep27184-s1.pdf]

Scientific Reports
Supplementary Material

Functional analysis of thioredoxin from the desert lichen-forming fungus, *Endocarpon pusillum* Hedwig, reveals its role in stress tolerance

Hui Li[1, 2] & Jiang-Chun Wei[1, *]

[1] State Key Laboratory of Mycology, Institute of Microbiology, Chinese Academy of Sciences, Beijing 100101, China;

[2] University of Chinese Academy of Sciences, Beijing 100049, China

*Corresponding author (email: weijc2004@126.com)

Table S1 Information of sequences from evolutionarily divergent organisms used in our comparative analysis

**Species**	**Similarity to EpTrx**	**Genbank number**
Arabidopsis thaliana	36.7%	NP_199112
Triticum aestivum	38.53%	AAL67139
Oryza sativa	35.78%	NP_001059069
Nicotiana tabacum	37.04%	Q07090
Aspergillus nidulans	53.21%	CBF90049
Neurospora crassa	42.45%	XP_962887
Saccharomyces cerevisiae	46.15%	NP_011725
Escherichia coli	32.65%	EFJ81602

Table S2 Primers designed for this study

**Primers**	**Sequence (5'-3')**	**Application**
pPIC9KTrx-F	CTACCATGGAATTCATGTCTGACACTGGAGTT	Clone to pPIC9K
pPIC9KTrx-R	ATAAGAATGCGGCCGCTCACTTGTGCTGCTCAATT	Clone to pPIC9K
pPIC3.5KTrx-F	GCGAATTCGCCACCATGTCTGACACTGGAGTT	Clone to pPIC3.5K
pPIC3.5KTrx-R	ATAAGAATGCGGCCGCTCACTTGTGCTGCTCAATT	Clone to pPIC3.5K
RT-Trx-F	TAACCTTGGAAGCAAACCCG	Real-time PCR
RT-Trx-R	TGTGCCACATCGCTGACCT	Real-time PCR
RT-EF1α-F	CCGCCATCGTCAAGATGGTT	Real-time PCR
RT-EF1α-R	CTATTTCTTCGAGGCCTTTTG	Real-time PCR
MTTrx-1-R	GTGGCAAAGGAGTCGAGGA	Site-directed mutation
MTTrx-2-F	TCCTCGACTCCTTTGCCAC	Site-directed mutation
pYES2Trx-F	CGGAATTCAACACAATGTCTGACACTGGAGTTCA	Clone to pYES2.0
pYES2Trx-R	CCGCTCGAGTCACTTGTGCTGCTCAATTG	Clone to pYES2.0
